# Thesis—Excisions Involving the Joints of the Upper Extremity

**Published:** 1871-10

**Authors:** W. W. Miner


BUFFALO

### Medical and Surgical Journal.

VOL. XI. OCTOBER, 1871. No. 3.

### Original Communications.

——— :o: ———

### Thesis.

ART I.—*Excisions Involving the Joints of the Upper Extremity.*—Reports of Illustrative Cases. By W. W. MINER, Class of 1870-71.

In presenting the following history of cases which embrace excision, as involving all the joints of the upper extremity in which excision is generally a desirable operation, I have the pleasure to say that all the cases here mentioned have come under my own observation, and that all except one of these cases have occurred during the past sixteen months, and in the practice of my uncle and preceptor, Dr. J. F. Miner. For permission to bring forward this report, as well as for clinical instruction upon the cases, it is proper for me to acknowledge my indebtedness to the surgeon I have already mentioned. The case involving the carpal articulations is taken from the practice of my father, Dr. D. W. Miner, through whom it was brought to my notice.

The limits of a thesis forbid the general consideration of the propriety of the operations herein described. I endeavor only to present in the reports of each of these cases some one or more points which is there given especial prominence and interest, and concerning which exact knowledge is not already possessed. In the case involving the inter-phalangeal articulation of the thumb, it may, perhaps, be found that some originality in the operation there performed does exist, or that some value in the illustration, afforded by an actually occurring case, renders its report worth presentation here. Without further delay permit me to call attention to the following cases:

I.—*Excision of the Humerus, including the Shoulder Joint.*

J. Packard, aged 22, living at No. 169 Cedar Street, Buffalo, while riding in a buggy, near Williamsville, on a hunting excursion, accidently discharged the contents of his shot-gun into his left shoulder and side. Arriving home at evening, his injury and condition was such that all operative interference was delayed until the next day. It was found that the upper third of the humerus had been completely shattered, and that the soft parts on the internal side of the arm were extensively lacerated, filled with shot, scorched and discolored by the discharge of powder. A wound six inches in length and three inches wide had been made in the direct line of the the intercostal space, and its depth was such as to expose plainly two ribs and also the intercostal muscle between them, which rose and fell with the motions of respiration. The periosteum of the outer surface of the exposed ribs had been carried away to a slight extent. Dr. Miner, in consultation with the family physician, Dr. Strong, and in the presence of several other physicians, decided that excision of the head of the humerus and of so much of the shaft as was required, should be undertaken.

I may here state that, although the patient was at the time of the injury in a condition of perfect health; still death seemed an almost necessary sequence. This was liable to occur from several causes, namely: from general shock, from perforation of the pleura, from pyæmia and from secondary hemorrhage. Never have I seen any similar case in which such imminence and multiform dangers threatened the patient. It seemed as though no operation but amputation could be of avail, or could be anything else than eminently hazardous. The only method by which fatal pyæmia could be avoided would appear to be by amputation, which would leave a clean surface as the site of the suppurative process. The statistics of cases of gun-shot injury to the head of the humerus as presented by the surgical reports of the War of the Rebellion, give 32.48 per cent. as the mortality after excisions of the head of the humerus, and 39.24 per cent. as the mortality after amputations at joint, thus giving the small difference of only 6.76 per cent. in favor of excisions.

Excision of the head with the upper and middle two-thirds of the humerus was in the present instance performed. The periosteum of the humerus was separated from the bone and not removed by the operation. A single handful of shot of about two ounces weight was removed from the tissue of the arm during the operation. It was not thought that then all the shot was removed. On the next day after the operation the patient was in such a state of depression that he did not rally to give any signs of recovery from shock until forty-eight hours after the operation was performed. Anodynes, stimulants and tonics, were used with the greatest care during the first three weeks from the operation, and were also continued during convalescence. Exfoliations of bone separated from the ribs on the third week after the injury. Antiseptic dressings, with careful and frequent cleansing of the parts, were, of course, the important means of local treatment. A straight splint was used to keep the parts in place and to allow of the convenient moving of the limb.

The patient gradually recovered, and this fact can be accounted for only on the ground that he was in perfect health at the time of the accident, and was very assiduously cared for afterwards. The injury was in all respects severe to the utmost extent. The last visit made the patient by Dr. Miner was on September 20th, three months after the injury. By November the patient had so nearly regained his strength as to present himself at the Winter clinic before the class of this institution. The result was as perfect as the injury was severe. The usefulness of the fore-arm is in every respect perfect. Very little motion of course can be made in the arm, which, however, shortened only one inch, while the extent of the excision was six inches. Three inches and one-half of bone have been replaced by periosteal reproduction; one and one-half inches are occupied by a ligamentous attachment between the joint and the excised extremity; this, with the shortening, accounts for the present condition of the arm. The observation made respecting the teaching of the case is, that a gun-shot injury of the most severe character does not increase the extent and danger of subsequent suppuration over that succeeding a simple contused wound, to such a degree as has been believed: again, the extent to which the periosteum, if uninjured, will reproduce bone is here finely illustrated.

CASE II.—*Excision of the Ulna involving the Elbow.*

Wm. J. Leech, aged 32, residing on Carroll Street, Buffalo, while employed as brakeman on the Lake Shore R. R., was, on the fifth day of November, 1869, caught between car couplings in such a manner as to crush the upper third of the ulna, and to lacerate to some considerable extent the soft parts on the posterior part of the fore-arm and immediately surrounding the comminuted ulna. The injury was received on the road some little distance from Dunkirk. The physicians who were called at Dunkirk advised immediate amputation of the arm. The patient preferred riding to Buffalo, where he might obtain further advice as to the necessity of amputation. Though the injury occasioned somewhat remarkable comminution of bone, and some considerable laceration of tissue, still it was found that the ulna was alone the seat of fracture, and that circulation and sensibility in the hand and forearm was not in any particular degree affected. The longitudinal opening in the integument was lengthened by incision so as to extend as far down as did the fractured bone. The upper and middle thirds of the ulna were removed by excision, while the radius was left intact. The limb was afterward placed upon an angular splint whose obliquity was varied as was necessary. Though the shock of the injury was very considerable, still the attempt at the preservation of the limb gave the patient courage, which was a valuable adjurant in his recovery. Carbolic acid water dressings were assiduously employed, and the cleansing of the parts with water was carefully and regularly attended to. Suppurative discharge was abundant, and to this, from the position of the wound on the posterior part of the forearm, there was afforded ready exit. Visits to the patient at his house were required for a period of six weeks, after which time he came regularly to the office, where the last dressing the case received was on the 29th day of December, only fifty-four days after the receipt of the injury. The result of the excision is a most satisfactory one. The motions of the fore-arm and hand are admirably retained. The man is now at work in a stove manufactory in this city, and his employer states that he is able to notice no difference in the efficiency of this workman from that of his fellows. The case goes to show that injury to the bony structure of a limb, though it involve two-thirds the extent of that bone and implicates its articular extremity, is of not so serious consequence as if the same extent of injury involved an equal extent of surrounding soft tissue. This conclusion was very strongly affirmed by a case of contusion of the soft parts of the fore-arm of the same extent as was that of fracture in the case already narrated which also was without co-extensive contusion of soft tissue. The patient with simple contusion and without fracture died, while that with fracture unaccompanied with co-extensive contusion was at no time very dangerously ill. The maxim which seems to be in process of adoption by surgeons is:—*Never amputate a limb for simple injury to its bony structure.*

CASE III.—*Excision of the Extremity of the Radius involving the Wrist joint.*

Joseph Humbert, aged 18 years, living at No. 56 Pratt Street, Buffalo, on the 22nd day of September, 1870, while working at the Union Iron Works, had a heavy iron weight fall upon the fore-arm of his right hand near its articulation with the wrist. The force of the blow was such as to crush the lower third of the radius into many fragments, and to so thoroughly contuse the muscular tissue as to fully expose the radial artery and nerve,as well as the extremities of the radius and ulna. The wound extended from the wrist-joint about four inches upward towards the elbow, being confined to the inner surface of the fore-arm. Dr. Green, who was first called to attend the case, requested Dr. Miner to advise as to the treatment to be instituted. Having induced anaesthesia it was found that the radial artery was not occluded, but that the circulation in both radial and ulna arteries was to a good degree intact. The principal feature which rendered doubtful what was the proper course of treatment was that the soft parts just above the wrist-joint were so severely contused that gangrene, or pyæmia seemed imminent to their retention.

However, excision of four inches of the comminuted extremity of the radius was made; the ulna was left of its full length although it had been more or less separated from its surrounding soft parts for nearly the whole of the lower third of its extent. In the operation considerable care was taken in removing thoroughly the numerous fragments of bone from the wound so as to lessen as much as could be possible the extent of the subsequent suppurative process. The limb was fastened upon a straight splint by means of bandages applied to the hand and to the upper part of the fore-arm. The injured parts were left entirely open, being dressed with carbolic acid water cloths. The suppuration which followed was, of course, of great extent, still the parts gradually took on healthy action, and in the space of about ten weeks were fully reproduced. In order to preserve motion at the wrist, the splint was removed at the end of the fourth week and the patient was enjoined to make use of passive motion of the wrist. As the patient was somewhat of an imbecile, no efforts whatever on his part could be induced. The splint was again applied to prevent the turning of the hand by contraction on the side of the excised bone. The patient now has a hand which presents a better appearance than an artificial one would be. It is of questionable utility however, as the patient has persisted in making no effort at motion whatever since the receipt of the injury. The case is quite remarkable, in that it shows that a crushing weight may fall upon the fore-arm at its junction with the wrist with such force as to break into fragments its bony structure without necessarily producing sufficient degree of contusion of the soft parts as to cause their destruction.

CASE IV.—*Excision of Bones of the Carpus.*

Patrick Donnehue, aged thirty-four, living at Ware, Mass., while tending a carding machine in a cotton mill, in December, 1869, received a severe crushing force upon the carpal and metacarpal bones of his right hand. The external wound was entirely upon the dorsal surface of the hand. Dr. D. W. Miner was called to the charge of the case. Amputation seemed to be inevitable. With the idea that secondary amputation might be made, and that by temporization, opportunity of observing the natural tendency of the injury might be gained, the hand was extended upon a straight splint, upon which it settled down like a plastic mass. Such few fragments of bone as were to be easily detached, were removed. Dressings of strong carbolic acid water were carefully and continuously applied for a period of six weeks. The suppuration which took place was of an active character, and the pus formed emitted a very strong and plentiful odor. The hand, however, after some three weeks healed over, and the result proves that a very useful member was preserved by the treatment which was pursued. The particular lesson given by the case is, that an injury of such severity as produce fracture of the carpal bones, and of such extent as to involve nearly all the articulating surfaces of the carpus, may not result in such inflammation or suppuration as to destroy the parts involved.

CASE V.—*Excision of the Extremity of the First Phalanx of the Thumb.*

Jacob Gilbert, 18 years of age, residing at No. 30 Hollister Street, Buffalo, on the 16th day of November, 1871, while attending a "Yankee Whittler," used for the manufacture of cigar boxes, inflicted a severe injury to the thumb of his right hand. On examination, it was found that the phalangeal articulation and extremity of the first phalanx of the thumb was shattered into several fragments. The integument and muscular tissue of the lateral surfaces of that bone were more or less completely severed. The tendons of the extensor and flexor longus pollicis were neither of them completely divided, though the utility of the latter was probably destroyed. The arterial supply to the extremity and last phalanx of the thumb was thought to be not altogether cut off, though it was doubtful whether the supply was adequate to sustain the vitality of that part. In the treatment of the case deliberation was required, notwithstanding the fact that injuries affecting the digital extremities have all been considered, until recently, as of very trifling importance. The injury was sufficiently severe to present the general indications for the operation of amputation. Had the injury been one involving either of the fingers instead of the thumb, amputation of the wounded extremity would even then have been made without great hesitation. Any one of the fingers can be dispensed with without seriously disabling the hand; the thumb, however, is indispensable in the performance of its common functions. But a very limited number of objects are to be grasped by the hand and fingers alone, and the grasp thus obtained is comparatively a very weak one, as is easily demonstrated. The ability to seize an object projecting towards one is only to be had through the means of two outwardly projecting extremities : this ability in the hand increases, to a certain extent, with the length of the finger and thumb. *A long thumb then, though it be a stiff one, is an organ of the greatest value.* Brevity of the thumb is accompanied with a corresponding abbreviation of the functional power of the hand. The course to be then pursued was decided accordingly. The ordinary operation of excision was performed upon the digital extremity of the first phalanx of the thumb. The loose fragments of the articulating surface were removed, and the sharp pointed extremity of bone smoothed off with bone forceps. The two phalanges were then left in contact with each other with the desire that firm anchylosis between them should take place. The principal incisions were closed with stitches. A carbolic acid, water dressing was loosely applied to the parts for about a week, when the purulent discharge already showed some abatement. A strip of firm adhesive plaster was then wrapped spirally about the thumb to serve as a splint to keep the bones in a correct line with each other, as they seemed inclined to unite at an angle. Daily cleansing of the parts with soap and water was performed by the patient, as the adhesive plaster splint formed no obstacle whatever to the examination and dressing of the granulating wound. By varying the tension brought to bear with with the adhesive plaster splint, the bones at length were brought into perfect line with each other, and complete anchylosis was obtained in six weeks from the time of the injury. The shortening which was caused is only one-half an inch, and, though motion in the last joint of the thumb is lost, that of every other muscle of the thumb is retained. In short, the result was equal to the most sanguine expectations.

Gross says that, "excision of the head of the phalanx of the thumb has sometimes been practiced," but I have nowhere been able to find this operation of excision of the inter-phalangeal articulation of the thumb instanced or described. Respecting the treatment of the phalangeal articulations, Gross continues to say—"It is never desirable to exsect any of the digital phalanges." It seems probable that the superior importance of this operation of excision in the phalangeal articulations of the thumb to the same operation in the phalanges of the fingers escaped notice or especial mention though Erichsen, in his latest edition, quite advocates conservatism in the treatment of injuries of the thumb.

However that may be, the result which has just been mentioned as occurring in the present instance is such as to determine unquestionably the eligibility of the operation which was here instituted.

